# Patient-reported outcome measures in central disorders of hypersomnolence: consensus of a sleep consortium/RARE-X expert working group

**DOI:** 10.1093/sleepadvances/zpag021

**Published:** 2026-02-13

**Authors:** Karmen Trzupek, Claire Wylds-Wright, Cynthia Kuan, Sleep Consortium Expert Working Group, Lynn Marie Trotti, Kiran Maski, Yves Dauvilliers, Luis Ortiz, Diego Mazzoti, Michelle Chadwick, Joshua Steinerman, Jennifer Gudeman, Deborah Hartman, Michael Doane, Suresh Kotagal, Fang Han, Murat Sincan, Emmanuel Mignot, Lindsay Jesteadt

**Affiliations:** Global Genes, 1012 14th Street NW, Washington, DC 20005, United States; Sleep Consortium, 14251 Ardel Drive, West Palm Beach, FL 33410, United States; Global Genes, 1012 14th Street NW, Washington, DC 20005, United States; Sleep Consortium, 14251 Ardel Drive, West Palm Beach, FL 33410, United States

**Keywords:** narcolepsy, pediatrics – narcolepsy, sleepiness

## Abstract

Central disorders of hypersomnolence (CDoH), including the primary hypersomnolence disorders of narcolepsy type 1 (NT1), narcolepsy type 2 (NT2), idiopathic hypersomnia (IH), and Kleine-Levin syndrome (KLS), as well as secondary hypersomnolence disorders, represent an underdiagnosed and under-treated population. Continuing advancements in understanding and treating CDoH rely on an understanding of the patient and caregiver experience. To address this need, a community-led, patient-owned online research study was launched by the nonprofit organizations Sleep Consortium and Global Genes, using the RARE-X research platform. An expert working group of stakeholders with expertise in hypersomnolence disorders, including clinicians, therapy developers, and patient advocates, was convened to identify key patient- and caregiver-reported clinical outcome measures essential for evaluating CDoH symptoms and impacts. These clinical outcome measures have been implemented as part of an online direct-to-patient study. The measures chosen by the Sleep Consortium Expert Working Group are presented here with the hope of supporting the standardization of clinical outcome assessments being used in CDoH research, especially for primary hypersomnolence disorders.

## Introduction

Central disorders of hypersomnolence (CDoH) are a group of sleep disorders characterized by pathologic daytime sleepiness [1]. The International Classification of Sleep Disorders, Third Edition, Text Revision (ICSD-3-TR) [2] classifies eight different CDoH: narcolepsy type 1 (NT1), narcolepsy type 2 (NT2), idiopathic hypersomnia (IH), Kleine-Levin syndrome (KLS), hypersomnia associated with a mental disorder, hypersomnia due to a medical disorder, hypersomnia due to a medication or substance, and insufficient sleep syndrome [2]. These disorders are further categorized into “primary” and “secondary” hypersomnolence disorders. The primary disorders include NT1, NT2, IH, and KLS, where excessive daytime sleepiness (EDS) and associated symptoms are intrinsic to the hypersomnolence disorder itself. The secondary disorders include hypersomnia due to a medical condition, hypersomnia due to a drug or substance, and insufficient sleep syndrome (ISS), where the hypersomnolence is secondary to other conditions or external factors or comorbid to mood disorders. This report will focus on the primary disorders NT1, NT2, IH, and KLS, where EDS is a direct manifestation of the hypersomnolence disorder.

Clinically, NT1 is marked by EDS and additional symptoms such as cataplexy, sleep paralysis, hallucinations, and disrupted nocturnal sleep. In contrast, IH can be characterized by at least 11 hours of sleep in a typical 24-hour period with severe sleep inertia. Although NT2 and IH can share a similar phenotype, differences can be seen on Multiple Sleep Latency Testing (MSLT), with NT2 patients having a mean sleep latency of at least 8 minutes and 2 or more sleep-onset REM periods [2]. KLS, notably, is episodic, with patients experiencing periods of extreme sleepiness and prolonged sleep lasting days to weeks [3].

Prevalence estimates for NT1, NT2, and IH have changed significantly over the last 20 years and are still debated. The overall prevalence of narcolepsy is estimated to be around 0.02–0.05 per cent of the general population [4]. While earlier studies suggested that 50–60 per cent of patients with narcolepsy experience episodes of cataplexy [4], some recent population-based studies have suggested that NT2 (without cataplexy) may be more common than NT1 [5, 6]. However, the definition of and exclusion criteria for NT2 vary in practice and can impact prevalence estimates significantly (input of Sleep Consortium Expert Working Group). For IH, data from the Wisconsin Sleep Cohort Study, which used both objective and subjective sleep data to identify probable cases, suggested that the prevalence of IH could be as high as 1.5 per cent in those impacted by sleep apnea, though this is widely known to be an overestimate in comparison to the general population [7]. Prior studies had estimated IH prevalence between 0.03 and 0.1 per cent, with variability due to diagnostic criteria and challenges in standardizing IH diagnosis [3]. The rarest condition, KLS, affects approximately 1 to 5 people per 1 million, making it exceedingly rare [8].

Despite advancements in understanding and treating CDoH in recent years [3], there is a pressing need for further research to better capture the patient and caregiver experience. Expanding this knowledge can inform updates to diagnostic criteria, enhance treatment strategies, and ultimately improve the quality of life (QoL) for patients and caregivers. Since CDoH conditions are rare, gathering detailed, meaningful data from individuals with these disorders and their caregivers is crucial. This approach will help identify the most significant symptoms and impacts, ensuring that interventions are tailored to the lived experiences of those affected by CDoH.

Sleep Consortium, a non-profit organization, was established to advance cutting-edge research, enhance disease understanding, and drive therapy development for people living with CDoH and related conditions. In collaboration with RARE-X (the research program of Global Genes, a non-profit organization), which offers a global survey platform for data-sharing and analysis to accelerate the development and approval of treatments for rare diseases, Sleep Consortium sought to establish a core set of patient-reported clinical outcome assessments (COAs) for CDoH. To achieve this, Sleep Consortium established an expert working group with global CDoH researchers to identify key patient- and caregiver-reported outcome measures, ensuring these assessments are easily accessible via online surveys and essential for evaluating CDoH symptoms and impacts. These COAs may help improve diagnostic criteria in the future by incorporating the patient perspective. We hope insights from these COAs will also guide future research and support therapy development. The primary goal of incorporating measures into the RARE-X platform for assessing key CDoH symptoms and impacts is to establish a strong baseline of data. This baseline will serve as a valuable starting point for further exploration of the CDoH disease experience. Additionally, it aims to provide accessible and comprehensive information on CDoH conditions to a broad audience, including patients, their families, clinicians, and researchers. This will facilitate a deeper understanding and drive further research and treatment advancements.

## Objective

The objective of this report is to summarize the collaborative efforts of Sleep Consortium and Global Genes and their selection of clinical outcome measures, specifically those reported by patients or caregivers. These have been implemented on the RARE-X research platform, as part of a patient-led research study called the DREAMS Portal. These measures are designed to enable affected individuals to contribute data on the lived experience of these conditions, which will provide important insights into the symptoms and impacts of CDoH that are most significant and meaningful to the patient population. By establishing these measures, the partnership aims to ensure a comprehensive understanding of CDoH conditions to enhance research, diagnosis, and therapeutic strategies.

## Methods

**Figure 1:**
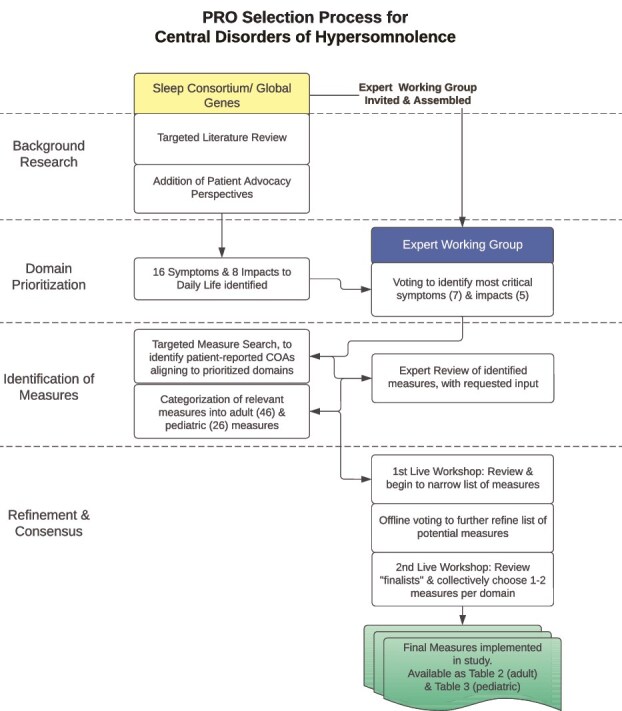
Process for selecting patient-reported COA measures for CDoH.

An overview of the process for selecting patient-reported COA measures for CDoH is provided as Figure 1. First, a targeted landscape review of CDoH was conducted to identify frequently reported symptoms and impacts of CDoH. Primary sources included peer-reviewed journal articles identified through PubMed, with supplementary clinical information from the National Institute of Neurological Disorders and Stroke on narcolepsy [9], the Stanford Center for Narcolepsy [10], and a review of sleep disorders by the Mayo Clinic [11]. A supplementary Google Scholar search was performed using terms such as “symptoms/impacts of Narcolepsy Type 1/Narcolepsy Type 2/Idiopathic Hypersomnia/Kleine Levin Syndrome.”

Patient-centered input was drawn from the Narcolepsy Summary Report from the US Food and Drug Administration (FDA) Voice of the Patient on Narcolepsy [12], the Narcolepsy & Idiopathic Hypersomnia Report from the FDA Patient-Led Listening Session [13], and patient stories and information sheets developed by relevant patient advocacy organizations including the Hypersomnia Foundation, the Sleep Consortium, and Project Sleep.

A working group of global experts in hypersomnolence was convened to: (1) review and prioritize the CDoH symptoms and impacts identified through the landscape analysis, and (2) review and later choose specific patient-reported clinical outcome measures for implementation as part of a community-driven online research study in CDoH. This working group consisted of 14 experts with a wide range of areas of expertise, including researchers (75%), clinicians (33%), industry partners (33%), patient advocates (25%), individuals living with CDoH (17%), and caregivers of individuals living with CDoH (17%); the full list of participants and affiliations is listed in Table 1. Fifty percent of the expert working group members currently work in academia. Working meetings and offline voting occurred over a 5-month period, from October 2023 to March 2024.

**Table 1:** Sleep consortium expert working group makeup

Name	Professional role	Affiliation and country
Michelle Chadwick	Founder/ Director; Patient Advocate	Sleep Disorders Australia & hypersomnolence Australia, nonprofit; Australia
Yves Dauvilliers, MD, PhD	Professor, Neurology and Physiology; Head of Clinical and Research Activity for the Sleep Laboratory	University of Montpellier, France
Michael Doane, PhD	Senior Director, Head of Health Economics and Outcomes Research (HEOR)	Alkermes; United States
Jennifer Gudeman, PharmD	Vice President, Medical and Clinical Affairs	Avadel Pharmaceuticals; United States
Fang Han, MD	Professor, Director of Center for Sleep Research & Center for Sleep Medicine	Peking University Sleep Research Center; China
Deborah Hartman, PhD	Global Scientific Head, Orexin Program	Centessa Pharmaceuticals; United States
Lindsay Jesteadt	Co-Founder, CEO; Patient Advocate	Sleep Consortium & Hypersomnia Foundation, 501(c)(3); United States
Suresh Kotagal, MD	Professor Emeritus, Department of Neurology; Clinical Sleep Research	Mayo Clinic; United States
Kiran Maski, MD, MPH	Associate Professor of Neurology, Div Sleep Medicine; Child Neurologist & Sleep Medicine Specialist.	Harvard University; Boston Children’s Hospital; United States
Diego Mazzoti, PhD	Associate Professor, Medical Informatics; Sleep Research focus	University of Kansas Medical Center; United States
Emmanuel Mignot, MD, PhD	Craig Reynolds, Professor of Sleep Medicine; Director of the Stanford Center for Narcolepsy	Stanford University; United States
Luis Ortiz, MD	Assistant Professor of Pediatrics, Sleep Center	Johns Hopkins University; United States
Murat Sincan, MD, FAMIA	Director of Health Informatics; Assistant Professor of Medicine	Essex Management LLC; Sanford School of Medicine; United States
Joshua Steinerman, MD	Vice President, Therapeutic Area Head, Neuroscience	Jazz Pharmaceuticals; United States
Lynn Marie Trotti, MD, MSc	Director, Emory Sleep Center; Associate Professor, Neurology	Emory University; United States
Claire Wylds-Wright	Co-Founder, CXO; Patient Advocate	Sleep Consortium & Hypersomnia Foundation, 501(c)(3); United States

Listed alphabetically by last name.

The most frequently identified symptoms (*n* = 16) and impacts (*n* = 8) of CDoH conditions were collected by the Global Genes team using a literature search and discussion with patient advocacy group leaders and shared with the CDoH working group. Experts were tasked with ranking the 16 symptoms (from 1 = most important to 16 = least important) and eight impacts (1 = most important to 8 = least important). There was a response rate of 86 per cent (12 of the 14 experts responded).

To identify patient-reported outcome measures (PROs) assessing each prioritized symptom or impact, a targeted search was conducted to first identify measures used in clinical studies focused on any of the primary CDoH conducted from 2018 to 2023, including those listed on Clinicaltrials.gov. Additional targeted searches were then performed on NHLBI.gov and NIH.gov for sleep disorders, ePROVIDE: an online repository of COAs, and healthmeasures.net (PROMIS and Neuro-QoL tools). Relevant patient advocacy organizations, including Sleep Consortium, Hypersomnia Foundation, and Kleine Levin Syndrome Foundation, as well as the members of the CDoH Expert working group, were also consulted. Measures were categorized by the primary domain assessed.

In total, 46 measures assessing symptoms and impacts of CDoH in adults and 26 measures assessing symptoms and impacts of CDoH in children were reviewed. All measures were presented to the CDoH working group in the first workshop, and experts were tasked with selecting the most appropriate measure to assess each symptom/impact domain. A full list of measures discussed in the first workshop is available in Supplementary Table 1.

For domains where members of the expert working group suggested or supported more than one potential measure, the group provided input using both live workshops and offline private input to narrow and then choose measures for inclusion in the study. The live meetings were used to review and discuss the strengths and weakness of each measure, comparing number of items, response option types, severity/frequency queried, target population (i.e. adult or pediatric), report method (i.e. self- or caregiver-report), population developed and validation details, development procedure (i.e. by experts and/or patients), and psychometric properties when available (e.g. internal consistency, construct validity, test–retest reliability, known group validity, responsiveness across time). Within each domain, the top measures (based on voting and suggestions) were selected and mapped to examine the level of overlap between similar measures.

A total of 54 measures were subsequently discussed in the second workshop, including 30 measures assessing symptoms of CDoH, 18 measures assessing impacts of CDoH, and six measures assessing other domains of interest. The expert working group discussed the profile, pros, and cons of each measure during the workshop to choose one or two measures for each domain that could potentially be implemented on the RARE-X survey platform for CDoH conditions. High-quality measures that assess more than one symptom or impact domain were well received by the expert working group, as that efficiency reduces participant burden and improves data completion.

## Results

**Figure 2:**
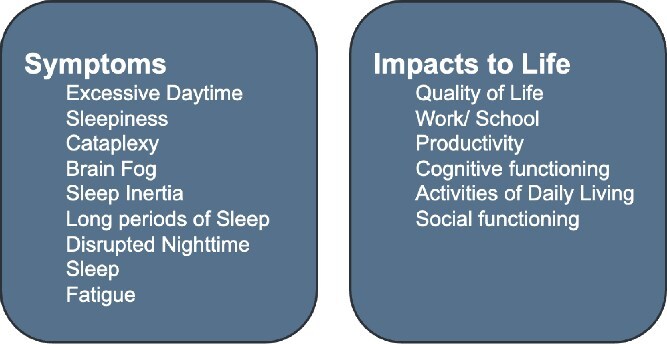
Prioritized symptoms and impacts to life to address in patient-focused hypersomnolence research. Symptoms of CDoH were chosen first. Impacts to life resulting from CDoH were chosen independently.

The expert working group prioritized seven symptoms, including EDS, cataplexy, brain fog, sleep inertia/sleep drunkenness, long periods of sleep, disrupted nighttime sleep/fragmented sleep/insomnia, and fatigue. Five impacts to life were prioritized: QoL, work or school productivity, cognitive functioning, activities of daily living (ADLs), and social functioning (Figure 2).

Details and brief descriptions of the measures chosen by consensus are available in Table 2 (for adults) and Table 3 (for children). Because no PROs were identified that quantify long periods of sleep, either through landscaping or expert consultation, this domain will be addressed in a future phase of work. Potential approaches include the incorporation of an externally collected sleep diary, sleep monitoring data using at-home devices, and curated clinical sleep study data, or a combination of these.

The chosen measures were implemented as part of the Sleep Consortium DREAMS Portal study, to enable the patient community to collect data on the symptoms and impacts to daily living in individuals affected with CDoH.

## Impact

Capturing robust, research-grade patient data requires the use of consistent methods of measurement, which prompted the collaborative effort described in this paper to choose patient-reported COA measures that can be compared between affected individuals and across studies.

The choice of these measures was critical to the implementation of the Sleep Consortium DREAMS Portal study, which aims to engage the CDoH patient community to: (1) improve knowledge and understanding about CDoH, (2) decrease the time to accurate diagnosis, and (3) accelerate the development of meaningful therapies & treatment options. The current management of patients with hypersomnolence disorders follows clinical guidance developed by physicians, based on a combination of their own experiences managing patients and available scientific publications. The DREAMS Portal study will use data provided directly by affected patients and caregivers using the COAs selected to create a dataset that reflects the patient experience- a critical component in developing informed, patient-centered care practices.

**Table 2:** Measures selected for adults with hypersomnolence

Adult		
Domain	Measure	Advantage of measure
EDS	Epworth Sleepiness Scale (ESS) [14]	Extensively validated in CDoH and used in clinical trials; “gold standard” of measuring EDS
	IH Severity Scale [15]	Assesses severity, frequency, and related impact; covers multiple domains of interest; extensively validated in CDoH and used in clinical trials
Cataplexy	Narcolepsy Severity Scale [16]	Assesses severity, frequency, and related impact; covers multiple domains of interest; extensively validated in CDoH and used in clinical trials
Brain fog	PROMIS Cognitive Function 8a [17, 18]	Brief, covers important elements of brain fog
Sleep inertia	IH Severity Scale [15]	(see above)
Disrupted nighttime sleep	Narcolepsy Severity Scale [16]	(see above)
Fatigue	Flinders Fatigue Scale (FSS) [19, 20]	Brief can distinguish between fatigue and sleepiness
QoL	Short Form 36 (SF-36) [21–23]	Extensively used in many research fields and validated in sleep disorders; useful for cross-disorder research
Work/school productivity	WHODAS 2.0: Life Activities Work/School domain [24, 25]	Brief and covers important elements; useful for cross-disorder research
ADLs	FOSQ-10 [26]	Assesses impacts specific to sleep
Cognitive functioning	WHODAS 2.0: Understanding and Communication domain [24]	Brief, covers multiple symptoms relevant to CDoH, useful for cross-disorder research
Social functioning	PROMIS Ability to participate in social roles and activities 8a [27]	Brief but covers important issues related to social functioning that are frequently impacted by sleep disorders

CDoH: central disorders of hypersomnolence; PROMIS: Patient-Reported Outcomes Measurement Information System; WHODAS: World Health Organization Disability Assessment Schedule.

**Table 3:** Measures selected for children with hypersomnolence

Pediatric		
Domain	Measure	Advantage of measure
EDS	Epworth Sleepiness Scale for Children and Adolescents (ESS-CHAD) [28–30]	Brief, covers multiple occasions, has been extensively validated in CDoH, and used in clinical trials
	PROMIS Pediatric Sleep Impairment 8a [31]	Brief, validated in sleep disorders
Cataplexy	Pediatric Narcolepsy Severity Scale (NSS-P) [32]	Assesses severity, frequency, and related impact; covers multiple domains of interest; extensively validated in CDoH and used in clinical trials
Sleep inertia	Pediatric Daytime Sleepiness Scale [33]	Brief and covers important symptoms relevant to CDoH
Disrupted nighttime sleep	PROMIS Pediatric Sleep Disturbance 8a [31]	Brief, validated in sleep disorders
	Pediatric Hypersomnolence Survey [34]	Brief and covers important symptoms relevant to CDoH
	NSS-P [32]	(see above)
Fatigue	PROMIS Pediatric Fatigue 10a [35]	Brief, useful for cross-disorder research
Work/school absenteeism and productivity	WHODAS-Child: Life Activities domain [36]	Brief and covers important elements; useful for cross-disorder research
Cognitive functioning	WHODAS-Child: Understanding and Communication domain [36]	Brief, covers multiple symptoms relevant to CDoH; useful for cross-disorder research

CDoH: central disorders of hypersomnolence; PROMIS: Patient-Reported Outcomes Measurement Information System; WHODAS: World Health Organization Disability Assessment Schedule.

The diagnosis of hypersomnolence disorders is challenged by symptom overlap between conditions, an inadequate understanding of the underlying pathophysiology, and lack of access to specialists and objective testing. The diagnosis of IH can be particularly difficult to obtain, given that there are many causes of sleepiness and long sleep duration, no definitive biomarkers for IH, and conflicting practices about which disorders must be ruled out before making the diagnosis[37–39]. An additional barrier to the timely diagnosis of CDoH is that affected individuals often do not perceive themselves to have a medical condition and may not search for a diagnosis until their symptoms are severe or greatly impacting their lives [40]. Multiple studies have reported average delays in diagnosis of narcolepsy and IH ranging from 9 to 15 years [41, 42]. By inviting individuals with symptoms associated with hypersomnolence to participate in an online study, we aim to collect longitudinal data that can be used to improve the recognition of these conditions at an earlier stage and shorten this diagnostic journey.

Despite the burden that sleep-related disorders have on every aspect of health and daily life, affected individuals have few good treatment options. Treatment for hypersomnolence conditions, while shown to be beneficial as measured through PROs such as the IHSS, does not fully resolve symptoms [42]. Patient data collected by the Hypersomnia Foundation indicate that most individuals with IH who are on treatment continue to experience persistent daily EDS, difficulty awakening, loss of productivity, cognitive symptoms, and memory and attention deficits [42].

Little data is available to compare treatments in CDoH, given the lack of side-by-side clinical trials and the wide use of off-label medications [39]. This creates a challenge for therapy developers, who are unable to correlate outcomes with standard-of-care treatments. The development of therapies requires access to data related to the type, severity, and progression of symptoms, comorbidities such as depression and cardiac disease, and medication usage [43, 44]. The Sleep Consortium’s patient-led central database will enable affected individuals to provide therapy developers access to this data, in addition to quantitative data related to QoL and impact to daily activities of living.

Multiple studies, both observational and interventional, are in process or development to address these issues and improve the QoL for those living with hypersomnolence. We hope that sharing the outcome of this working group will help to standardize the way that the patient experience is measured and used to contribute to disease understanding and the development of meaningful treatments.

## Limitations

We cannot rule out the possibility that the COA measures chosen by this expert working group may be biased by the composition of the expert working group, which includes several experts who serve on the Scientific Advisory Board of Sleep Consortium on a volunteer basis. Additionally, most of the working group members are from the United States and may represent an overall bias toward measures available in English.

Our findings suggest several needs in terms of assessing CDoH. First, measures assessing symptoms and impacts of CDoH in pediatric populations fall short of those available for adult populations. Multiple commonly used CDoH-specific measures only have adult versions. As a significant number of CDoH cases are children and adolescents, measures should be developed or adapted to target pediatric patients with CDoH. Second, most existing CDoH-specific measures focus primarily on symptoms. All but one of the measures chosen to quantify the impacts of hypersomnolence disorders (QoL, work/ school productivity, cognitive functioning, and social functioning) were developed for disorders other than CDoH or sleep disorders. The exception is the Functional Outcomes of Sleep Questionnaire (FOSQ)-10, developed specifically to address the impact of sleepiness on the ability to conduct daily activities. Additional disease-specific measures assessing the impact of hypersomnolence disorders- especially on cognitive functioning, work/school productivity, and social functioning- could assist in better understanding and reducing patient burdens. Third, additional research is needed to validate relevant measures in patient populations with sleep disorders and CDoH specifically. Lastly, although measures to address caregiver burden in those who provide support for individuals living with CDoH were not addressed in this report, assessing caregiver burden would also be important to capture the CDoH disease experience more fully.

## Supplementary Material

Publication_SleepAdvances_Supplementary_Materials_zpag021

## Data Availability

No new data were generated or analyzed in support of this research.
