# Supplementary material for: Patient-reported outcome measures in central disorders of hypersomnolence: consensus of a sleep consortium/RARE-X expert working group
Source: Sleep Adv. 2026 Feb 13;7(1):zpag021. doi: 10.1093/sleepadvances/zpag021 (PMC12978642; doi:10.1093/sleepadvances/zpag021)
Supplement: Publication_SleepAdvances_Supplementary_Materials_zpag021 [file publication_sleepadvances_supplementary_materials_zpag021.docx]

Patient-Reported Outcome Measures in Central Disorders of Hypersomnolence: Consensus of a Sleep Consortium/ RARE-X Expert Working Group

Supplementary Material

### *Karmen Trzupek^1^, Claire Wylds-Wright ^2^, Cynthia Kuan^1^, Sleep Consortium Expert Working Group^3^, Lindsay Jesteadt^2^*

### ^1^Global Genes, 1012 14th Street NW, Washington, DC 20005, USA

^2^Sleep Consortium, 14251 Ardel Drive, West Palm Beach, FL 33410, USA

### ^3^The Sleep Consortium Expert Working Group was convened to support this initiative, and includes the following experts in disorders of hypersomnia:

Lynn Marie Trotti; Kiran Maski; Yves Dauvilliers; Luis Ortiz; Diego Mazzoti; Michelle Chadwick; Joshua Steinerman; Jennifer Gudeman; Deborah Hartman; Michael Doane; Suresh Kotagal; Fang Han; Murat Sincan; Emmanuel Mignot

Corresponding author:

Karmen Trzupek

karmen.trzupek@globalgenes.org

# Supplementary Table 1. Clinical Outcome Assessment Measures (COAs) Considered and Chosen by Sleep Expert Working Group

| **Domain** | **Measures Initially Considered** | **Measures Selected for Discussion** | **Measures Chosen for Study Inclusion** |
| --- | --- | --- | --- |
| Excessive daytime sleepiness (EDS) | Adult measures:   - Epworth Sleepiness Scale^1, 2^ - Hypersomnia Severity Index^3, 4^ - Karolinska Sleepiness Scale^5^ - Narcolepsy Severity Scale^6^ - Idiopathic Hypersomnia Severity Scale^7^ - PROMIS Sleep-Related Impairment- 8a^8^ - SCOPA-Sleep^9^ - Stanford Sleep Disorders Questionnaire, Section VI: Symptoms of Sleepiness (can also be used in pediatric population) ^10^ - Stanford Sleepiness Scale^11^ - Ullanlinna Narcolepsy Scale^12^ - ZOGIM Alertness Scale^13^   Pediatric measures:   - Epworth Sleepiness Scale for Children and Adolescents^14, 15^ - Children’s Report of Sleep Patterns^16^ - Pediatric Narcolepsy Severity Scale^17^ - PROMIS Pediatric Sleep-Related Impairment 8a^18^ - Pediatric Daytime Sleepiness Scale^19^ - Pediatric Hypersomnolence Survey^20^ | Adult measures:   - Epworth Sleepiness Scale - Narcolepsy Severity Scale - Idiopathic Hypersomnia Severity Scale   Pediatric measures:   - Epworth Sleepiness Scale for Children and Adolescents - PROMIS Pediatric Sleep-Related Impairment 8a - Pediatric Daytime Sleepiness Scale | Adult measures:   - Epworth Sleepiness Scale - Idiopathic Hypersomnia Severity Scale   Pediatric measures:   - Epworth Sleepiness Scale for Children and Adolescents - PROMIS Pediatric Sleep-Related Impairment 8a |
| Cataplexy | Adult measures:   - Cataplexy Emotional Trigger Questionnaire^21^ - Swiss Narcolepsy Scale^22^ - Narcolepsy Severity Scale^6^ - Patient Global Impression of Severity and Patient Global Impression of Change^23^   Pediatric measures:   - Pediatric Hypersomnolence Survey^20^ - Pediatric Narcolepsy Severity Scale ^17^ - Patient Global Impression of Severity and Patient Global Impression of Change^23^ | Adult measures:   - Swiss Narcolepsy Scale - Narcolepsy Severity Scale - Patient Global Impression of Severity and Patient Global Impression of Change   Pediatric measures:   - Pediatric Hypersomnolence Survey - Pediatric Narcolepsy Severity Scale - Patient Global Impression of Severity and Patient Global Impression of Change | Adult measures:   - Narcolepsy Severity Scale   Pediatric measures:   - Pediatric Narcolepsy Severity Scale |
| Brain fog | Adult measures:   - Brain Fog Scale^24^ - PROMIS Cognitive Function 8a^25^ | Adult measures:   - Brain Fog Scale - PROMIS Cognitive Function 8a | Adult measures:   - PROMIS Cognitive Function 8a   Pediatric measures will be assessed via the measures included in the Cognitive Function/ Fatigue domains. |
| Long periods of sleep | No measure identified |  |  |
| Sleep inertia/sleep drunkenness | Adult measures:   - Sleep Inertia Questionnaire^26^ - Idiopathic Hypersomnia Severity Scale^7^   Pediatric measures:  No measure was found to specifically assess sleep inertia in pediatric population, but sleep inertia is addressed as a component of these measures:   - Pediatric Hypersomnolence Survey^20^ - Pediatric Daytime Sleepiness Scale^19^ - School Sleep Habits Survey^27^ | Adult measures:   - Idiopathic Hypersomnia Severity Scale   Pediatric measures:   - Pediatric Hypersomnolence Survey - Pediatric Daytime Sleepiness Scale - School Sleep Habits Survey | Adult measures:   - Idiopathic Hypersomnia Severity Scale   Pediatric measures:   - Pediatric Daytime Sleepiness Scale - School Sleep Habits Survey   *The full measure will be administered; these specific items were the ones assessing this domain. |
| Disrupted nighttime sleep/ fragmented sleep/ insomnia | Adult measures:   - Global Sleep Assessment Questionnaire^28^ - Insomnia Severity Index^29^ - Medical Outcomes Study Sleep Scale^30, 31^ - Munich Parasomnia Screening^32^ - Pittsburgh Sleep Quality Index^33^ - Narcolepsy Severity Scale^6^ - PROMIS Sleep Disturbance 8b^8^ - SCOPA-Sleep^9^ - Stanford Sleep Disorders Questionnaire Section V: Symptoms of insomnia (can also be used in pediatric population)^10^ - Sleep Quality Scale^34^   Pediatric measures:   - Brief Infant Sleep Questionnaire-Revised^35^ - Children’s Sleep Habits Questionnaire^36^ - Children’s Report of Sleep Patterns - Sleep pattern domain and Insomnia/Parasomnia Domain^16^ - PROMIS Sleep Disturbance 8a^18^ - Sleep Disturbance Scale for Children^37^ - Sleep Disturbances in Children with Severe Psychomotor Impairment^38^ | Adult measures:   - Stanford Sleep Disorders Questionnaire Section V: Symptoms of insomnia - PROMIS Sleep Disturbance - Insomnia Severity Index - Pittsburgh Sleep Quality Index - Narcolepsy Severity Scale   Pediatric measures:   - PROMIS Pediatric Sleep Disturbance 8a - Pediatric Hypersomnolence Survey | Adult measures:   - Stanford Sleep Disorders Questionnaire Section V: Symptoms of insomnia - Narcolepsy Severity Scale   Pediatric measures:   - PROMIS Pediatric Sleep Disturbance 8a - Pediatric Hypersomnolence Survey - Pediatric Narcolepsy Severity Scale |
| Fatigue | Adult measures:   - Fatigue Assessment Scale^39^ - Fatigue Severity Scale^40^ - Flinders Fatigue Scale^41, 42^ - Multidimensional Fatigue Inventory^43^ - PedsQL Multidimensional Fatigue Scale – Adult version^44^ - SF-36 (vitality/energy subscale)^45,46^   Pediatric measures:   - PedsQL Multidimensional Fatigue Scale – Pediatric version^47^ - PROMIS Pediatric Fatigue 10a^48^ | Adult measures:   - Flinders Fatigue scale   Pediatric measures:   - PROMIS Pediatric Fatigue 10a - Pediatric Hypersomnolence Survey | Adult measures:   - Flinders Fatigue scale   Pediatric measures:   - PROMIS Pediatric Fatigue 10a |
| Quality of life | Adult measures:   - Pediatric Quality of Life Inventory (PedsQL) Generic Core Scales- Adult version^49^ - PROMIS 29^50^ - SF-36^45,46^   Pediatric measures:   - KIDSCREEN (10-item version)^51^ - Pediatric Quality of Life Inventory (PedsQL) Generic Core Scales -Pediatric version^49^ | Adult measures:   - SF-36   Pediatric measures:   - KIDSCREEN (10-item version) | Adult measures:   - SF-36   Pediatric measures:   - None felt to be applicable enough to recommend at this time. |
| Work/school productivity | Adult measures:   - Pediatric Quality of Life (PedsQL) Work domain^49^ - WHODAS 2.0 Life Activities Work domain^52, 53^ - WHO Health and Work Performance Questionnaire^52, 53^ - Work Productivity and Activity Impairment Questionnaire: General Health^54^ - Functional Outcomes of Sleep Questionnaire- 30 item version^55^ - Work and Social Adjustment Scale – Adult version^56^   Pediatric measures:   - Pediatric Quality of Life (PedsQL) Study domain^49^ - WHODAS 2.0 Life Activities School domain^52, 53^ - Work and Social Adjustment Scale – Youth version^56^ | Adult measures:   - WHODAS 2.0 Life Activities Work domain - Functional Outcomes of Sleep Questionnaire- 30 item version   Pediatric measures:   - WHODAS 2.0 Youth version Life Activities School domain | Adult measures:   - WHODAS 2.0 Life Activities Work domain   Pediatric measures:   - WHODAS 2.0 Youth version Life Activities domain |
| Activities of daily living | Adult measures:   - Functional Outcomes of Sleep Questionnaire – 30 item version^55^ - Functional Outcomes of Sleep Questionnaire – 10 item version^55^ - WHODAS 2.0 Life Activities Household domain – Adult version^52,53^   Pediatric measures:   - WHODAS 2.0 Life Activities Household domain – Pediatric version^52,53^ | Adult measures:   - Functional Outcomes of Sleep Questionnaire – 10 item - Functional Outcomes of Sleep Questionnaire – 30 item   Pediatric measures:   - WHODAS 2.0 Youth version Life Activities Household domain – Pediatric version | Adult measures:   - Functional Outcomes of Sleep Questionnaire – 10 item   Pediatric measures:   - None. The working group decided to address this domain through other measures chosen for study inclusion. |
| Cognitive functioning | Adult measures:   - PROMIS Cognitive Function 8a^25^ - PROMIS Cognitive Function 6a^25^ - WHODAS 2.0 Understanding and communication domain – Adult version^52, 53^ - Brain Fog Scale^24^   Pediatric measures:   - PROMIS Cognitive Function 7a^25^ - WHODAS 2.0 Understanding and communication domain – Pediatric version^52, 53^ | Adult measures:   - PROMIS Cognitive Function 6a - WHODAS 2.0 Understanding and communication domain – Adult version - Brain Fog Scale   Pediatric measures:   - PROMIS Cognitive Function 7a - WHODAS 2.0 Youth version Understanding and communication domain | Adult measures:   - WHODAS 2.0 Understanding and communication domain – Adult version - Note: The working group recognized the inadequacy of the WHODAS to fully capture brain fog in hypersomnolence & recommended the consideration of developing a new measure.   Pediatric measures:   - WHODAS 2.0 Youth version Understanding and communication domain |
| Social functioning | Adult measures:   - PROMIS Ability to Participate in Social Roles and Activities 8a^57^ - PROMIS Ability to Participate in Social Roles and Activities 6a^57^   Pediatric measures:   - KIDSCREEN 27^51^ | Adult measures:   - PROMIS Ability to Participate in Social Roles and Activities 8a   Pediatric measures:   - KIDSCREEN 27 | Adult measures:   - PROMIS Ability to Participate in Social Roles and Activities 8a   Pediatric measures:   - None felt to be applicable enough to recommend at this time. |

# References

1. Pecotic, R., Dodig, I. P., Valic, M., Ivkovic, N., & Dogas, Z. (2012). The evaluation of the Croatian version of the Epworth sleepiness scale and STOP questionnaire as screening tools for obstructive sleep apnea syndrome. Sleep Breath, 16(3), 793-802. doi:10.1007/s11325-011-0578-x
2. Johns, M. W. (1991). A new method for measuring daytime sleepiness: the Epworth sleepiness scale. *Sleep, 14*(6), 540-545. doi:10.1093/sleep/14.6.540
3. Fernandez-Mendoza, J., Puzino, K., Amatrudo, G., Bourchtein, E., Calhoun, S. L., Plante, D. T., & Kaplan, K. (2021). The Hypersomnia Severity Index: reliability, construct, and criterion validity in a clinical sample of patients with sleep disorders. Journal of clinical sleep medicine: JCSM: official publication of the American Academy of Sleep Medicine, 17(11), 2249–2256. https://doi.org/10.5664/jcsm.9426
4. Kaplan, K. A., Plante, D. T., Cook, J. D., & Harvey, A. G. (2019). Development and validation of the Hypersomnia Severity Index (HSI): A measure to assess hypersomnia severity and impairment in psychiatric disorders. Psychiatry research, 281, 112547. https://doi.org/10.1016/j.psychres.2019.112547
5. Akerstedt T, Gillberg M. (1990). Subjective and objective sleepiness in the active individual. International Journal of Neuroscience, 52, 29–37.
6. Dauvilliers, Y., Beziat, S., Pesenti, C., Lopez, R., Barateau, L., Carlander, B., . . . Jaussent, I. (2017). Measurement of narcolepsy symptoms: The Narcolepsy Severity Scale. *Neurology, 88*(14), 1358-1365. doi:10.1212/WNL.0000000000003787
7. Dauvilliers, Y., Evangelista, E., Barateau, L., Lopez, R., Chenini, S., Delbos, C., . . . Jaussent, I. (2019). Measurement of symptoms in idiopathic hypersomnia: The Idiopathic Hypersomnia Severity Scale. *Neurology, 92*(15), e1754-e1762. doi:10.1212/WNL.0000000000007264
8. Daniel J. Buysse, Lan Yu, Douglas E. Moul, Anne Germain, Angela Stover, Nathan E. Dodds, Kelly L. Johnston, Melissa A. Shablesky-Cade, Paul A. Pilkonis, Development and Validation of Patient-Reported Outcome Measures for Sleep Disturbance and Sleep-Related Impairments, Sleep, Volume 33, Issue 6, June 2010, Pages 781–792, https://doi.org/10.1093/sleep/33.6.781
9. Marinus, J., Visser, M., van Hilten, J. J., Lammers, G. J., & Stiggelbout, A. M. (2003). Assessment of sleep and sleepiness in Parkinson disease. Sleep, 26(8), 1049–1054. https://doi.org/10.1093/sleep/26.8.1049
10. Douglass, A. B., Bornstein, R., Nino-Murcia, G., Keenan, S., Miles, L., Zarcone, V. P., Jr, Guilleminault, C., & Dement, W. C. (1994). The Sleep Disorders Questionnaire. I: Creation and multivariate structure of SDQ. Sleep, 17(2), 160–167. https://doi.org/10.1093/sleep/17.2.160
11. Hoddes, E., Zarcone, V., Smythe, H., Phillips, R. and Dement, W.C. (1973), Quantification of Sleepiness: A New Approach. Psychophysiology, 10: 431-436. https://doi.org/10.1111/j.1469-8986.1973.tb00801.x
12. Hublin, C., Kaprio, J., Partinen, M., Koskenvuo, M., & Heikkila, K. (1994). The Ullanlinna narcolepsy scale: validation of a measure of symptoms in the narcoleptic syndrome. Journal of Sleep Research, 3, 52–59.
13. Shapiro, C. M., Auch, C., Reimer, M., Kayumov, L., Heslegrave, R., Huterer, N., Driver, H., & Devins, G. M. (2006). A new approach to the construct of alertness. Journal of psychosomatic research, 60(6), 595–603. https://doi.org/10.1016/j.jpsychores.2006.04.012
14. Wang, Y. G., Benmedjahed, K., Lambert, J., Evans, C. J., Hwang, S., Black, J., & Johns, M. W. (2017). Assessing narcolepsy with cataplexy in children and adolescents: development of a cataplexy diary and the ESS-CHAD. *Nat Sci Sleep, 9*, 201-211. doi:10.2147/NSS.S140143
15. Wang, Y. G., Menno, D., Chen, A., Steininger, T. L., Morris, S., Black, J., . . . Johns, M. W. (2022). Validation of the Epworth Sleepiness Scale for Children and Adolescents (ESS-CHAD) questionnaire in pediatric patients with narcolepsy with cataplexy aged 7-16 years. *Sleep Med, 89*, 78-84. doi:10.1016/j.sleep.2021.11.003
16. Meltzer, L. J., Avis, K. T., Biggs, S., Reynolds, A. C., Crabtree, V. M., & Bevans, K. B. (2013). The Children's Report of Sleep Patterns (CRSP): a self-report measure of sleep for school-aged children. Journal of clinical sleep medicine : JCSM : official publication of the American Academy of Sleep Medicine, 9(3), 235–245. https://doi.org/10.5664/jcsm.2486
17. Barateau L, Lecendreux M, Chenini S, et al. Measurement of Narcolepsy Symptoms in School-Aged Children and Adolescents: The Pediatric Narcolepsy Severity Scale. Neurology. 2021; 97 (5).
18. Forrest, C. B., Meltzer, L. J., Marcus, C. L., de la Motte, A., Kratchman, A., Buysse, D. J., . . . Bevans, K. B. (2018). Development and validation of the PROMIS Pediatric Sleep Disturbance and Sleep-Related Impairment item banks. *Sleep, 41*(6). doi:10.1093/sleep/zsy054
19. Drake, C., Nickel, C., Burduvali, E., Roth, T., Jefferson, C., & Pietro, B. (2003). The pediatric daytime sleepiness scale (PDSS): sleep habits and school outcomes in middle-school children. *Sleep, 26*(4), 455-458. Retrieved from <https://www.ncbi.nlm.nih.gov/pubmed/12841372>
20. Maski, K., Worhach, J., Steinhart, E., Boduch, M., Morse, A. M., Strunc, M., . . . Sideridis, G. (2022). Development and Validation of the Pediatric Hypersomnolence Survey. *Neurology, 98*(19), e1964-e1975. doi:10.1212/WNL.0000000000200187
21. Moore, W. R., Silber, M. H., Decker, P. A., Heim-Penokie, P. C., Sikkink, V. K., Slocumb, N., Richardson, J. W., & Krahn, L. E. (2007). Cataplexy Emotional Trigger Questionnaire (CETQ)--a brief patient screen to identify cataplexy in patients with narcolepsy. Journal of clinical sleep medicine : JCSM : official publication of the American Academy of Sleep Medicine, 3(1), 37–40.
22. Sturzenegger, C., & Bassetti, C. L. (2004). The clinical spectrum of narcolepsy with cataplexy: a reappraisal. Journal of sleep research, 13(4), 395–406. https://doi.org/10.1111/j.1365-2869.2004.00422.x
23. Snyder, E. S., Tao, P., Svetnik, V., Lines, C., & Herring, W. J. (2021). Use of the single-item Patient Global Impression-Severity scale as a self-reported assessment of insomnia severity. Journal of sleep research, 30(1), e13141. https://doi.org/10.1111/jsr.13141
24. Debowska, A., Boduszek, D., Ochman, M., Hrapkowicz, T., Gaweda, M., Pondel, A., & Horeczy, B. (2024). Brain Fog Scale (BFS): Scale development and validation. Personality and Individual Differences, 216, Article 112427. https://doi.org/10.1016/j.paid.2023.112427
25. Grant L Iverson, Jacqueline M Marsh, Eric J Connors, Douglas P Terry, Normative Reference Values, Reliability, and Item-Level Symptom Endorsement for the PROMIS® v2.0 Cognitive Function-Short Forms 4a, 6a and 8a, Archives of Clinical Neuropsychology, Volume 36, Issue 7, October 2021, Pages 1341–1349, https://doi.org/10.1093/arclin/acaa128
26. Sung, E. R., Maness, C. B., Cook, J. D., Vascan, A. M., Moron, D., Saini, P., Rye, D. B., Plante, D. T., & Trotti, L. M. (2024). Validation and performance of the sleep inertia questionnaire in central disorders of hypersomnolence. Sleep medicine, 121, 352–358. https://doi.org/10.1016/j.sleep.2024.07.024
27. Wolfson, A. R., & Carskadon, M. A. (1998). Sleep schedules and daytime functioning in adolescents. *Child Dev, 69*(4), 875-887. Retrieved from <https://www.ncbi.nlm.nih.gov/pubmed/9768476>
28. Roth, T., Zammit, G., Kushida, C., Doghramji, K., Mathias, S. D., Wong, J. M., & Buysse, D. J. (2002). A new questionnaire to detect sleep disorders. Sleep medicine, 3(2), 99–108. https://doi.org/10.1016/s1389-9457(01)00131-9
29. Morin, C. M., Belleville, G., Bélanger, L., & Ivers, H. (2011). The Insomnia Severity Index: psychometric indicators to detect insomnia cases and evaluate treatment response. Sleep, 34(5), 601–608. https://doi.org/10.1093/sleep/34.5.601
30. Hays, R. D., & Stewart, A. L. (1992). Sleep measures. In A. L. Stewart & J. E. Ware (eds.), Measuring functioning and well-being: The Medical Outcomes Study approach (pp. 235-259), Durham, NC: Duke University Press.
31. Ware, J. E., Jr., & Sherbourne, C. D. (1992). The MOS 36-item short-form health survey (SF-36). I. Conceptual framework and item selection. *Med Care, 30*(6), 473-483. Retrieved from <https://www.ncbi.nlm.nih.gov/pubmed/1593914>
32. Fulda S, Hornyk M, Muller K, Cerny L, Beitinger PA, Wetter TC. Development and validation of the Munich Parasomnia screening (MUPS): a questionnaire for parasomnias and nocturnal behaviors. Somnologie 2008; 12: 56-65.
33. Buysse, D. J., Reynolds, C. F., Charles, F., Monk, T.H., Berman, S. R., & Kupfer, D. J. (1989). The Pittsburgh sleep quality index: a new instrument for psychiatric practice and research. Psychiatry Research, 28 (2), 193–213.
34. Yi, H., Shin, K., & Shin, C. (2006). Development of the sleep quality scale. Journal of sleep research, 15(3), 309–316. https://doi.org/10.1111/j.1365-2869.2006.00544.x
35. Sadeh, A. (2004). A brief screening questionnaire for infant sleep problems: validation and findings for an internet sample. Pediatrics, 113(6), e570–e577.
36. Owens JA, Spirito A, McGuinn M. The children’s sleep habits questionnaire (CSHQ): psychometric properties of a survey instrument for school-aged children. Sleep 2000, Dec 15;23(8):1043-51
37. Bruni, O., Ottaviano, S., Guidetti, V., Romoli, M., Innocenzi, M., Cortesi, F., & Giannotti, F. (1996). The sleep disturbance scale for children (SDSC): Construction and validation of an instrument to evaluate sleep disturbances in childhood and adolescence. Journal of Sleep Research, 5, 251–261.
38. Blankenburg, M., Tietze, A. L., Hechler, T., Hirschfeld, G., Michel, E., Koh, M., & Zernikow, B. (2013). Snake: the development and validation of a questionnaire on sleep disturbances in children with severe psychomotor impairment. Sleep medicine, 14(4), 339–351. https://doi.org/10.1016/j.sleep.2012.12.008
39. Michielsen, H. J., De Vries, J., & Van Heck, G. L. (2003). Psychometric qualities of a brief self-rated fatigue measure the fatigue assessment scale. Journal of Psychosomatic Research, 54, 345–352.
40. Krupp, L. B., LaRocca, N. G., Muir-Nash, J., & Steinberg, A. D. (1989). The fatigue severity scale: application to patients with multiple sclerosis and systemic lupus erythematosus. Archives of Neurology, 46, 1121–1123.
41. Cameron, K., Williamson, P., Short, M. A., & Gradisar, M. (2017). Validation of the Flinders Fatigue Scale as a measure of daytime fatigue. *Sleep Med, 30*, 105-112. doi:10.1016/j.sleep.2016.11.016
42. Gradisar, M., Lack, L., Richards, H., Harris, J., Gallasch, J., Boundy, M., & Johnston, A. (2007). The Flinders Fatigue Scale: preliminary psychometric properties and clinical sensitivity of a new scale for measuring daytime fatigue associated with insomnia. *J Clin Sleep Med, 3*(7), 722-728. Retrieved from <https://www.ncbi.nlm.nih.gov/pubmed/18198807>
43. Smets, E. M., Garssen, B., Bonke, B., & De Haes, J. C. (1995). The Multidimensional Fatigue Inventory (MFI) psychometric qualities of an instrument to assess fatigue. Journal of psychosomatic research, 39(3), 315–325. https://doi.org/10.1016/0022-3999(94)00125-o
44. Varni, J. W., & Limbers, C. A. (2008). The PedsQL Multidimensional Fatigue Scale in young adults: feasibility, reliability and validity in a University student population. Quality of life research : an international journal of quality of life aspects of treatment, care and rehabilitation, 17(1), 105–114. https://doi.org/10.1007/s11136-007-9282-5
45. Leger, D., Scheuermaier, K., Philip, P., Paillard, M., & Guilleminault, C. (2001). SF-36: evaluation of quality of life in severe and mild insomniacs compared with good sleepers. *Psychosom Med, 63*(1), 49-55. doi:10.1097/00006842-200101000-00006
46. Smith, I. E., & Shneerson, J. M. (1995). Is the SF 36 sensitive to sleep disruption? A study in subjects with sleep apnoea. *J Sleep Res, 4*(3), 183-188. doi:10.1111/j.1365-2869.1995.tb00167.x
47. Varni JW, Burwinkle TM, Szer IS. The PedsQL™ Multidimensional Fatigue Scale in pediatric rheumatology: reliability and validity. J Rheumatol. 2004 Dec;31(12):2494-500
48. Lai, J. S., Stucky, B. D., Thissen, D., Varni, J. W., DeWitt, E. M., Irwin, D. E., . . . DeWalt, D. A. (2013). Development and psychometric properties of the PROMIS((R)) pediatric fatigue item banks. Qual Life Res, 22(9), 2417-2427. doi:10.1007/s11136-013-0357-1
49. Varni JW, Burwinkle TM, Katz ER, et al. The PedsQL™ in pediatric cancer: reliability and validity of the Pediatric Quality of Life Inventory™ Generic Core Scales, Multidimensional Fatigue Scale, and Cancer Module. Cancer. 2002 Apr 1;94(7):2090-106
50. Devellis, R., DeWalt, D., Fries, J. F., Gershon, R., Hahn, E. A., Lai, J. S., Pilkonis, P., Revicki, D., Rose, M., … PROMIS Cooperative Group (2010). The Patient-Reported Outcomes Measurement Information System (PROMIS) developed and tested its first wave of adult self-reported health outcome item banks: 2005-2008. Journal of clinical epidemiology, 63(11), 1179–1194. <https://doi.org/10.1016/j.jclinepi.2010.04.011>
51. Ravens-Sieberer, U., Herdman, M., Devine, J., Otto, C., Bullinger, M., Rose, M., & Klasen, F. (2014). The European KIDSCREEN approach to measure quality of life and well-being in children: development, current application, and future advances. Quality of life research : an international journal of quality of life aspects of treatment, care and rehabilitation, 23(3), 791–803. https://doi.org/10.1007/s11136-013-0428-3
52. de Oliveira, A. C. S., Zacarias, L. C., de Souza, C. C. C., Bezerra, J. P. D. S., Viana-Junior, A. B., Sobreira-Neto, M. A., & Leite, C. F. (2024). Validity, reliability, and responsiveness of the Brazilian version of the instrument World Health Organization Disability Assessment Schedule (WHODAS 2.0) for individuals with obstructive sleep apnoea. Heart & lung : the journal of critical care, 67, 183–190. https://doi.org/10.1016/j.hrtlng.2024.05.010
53. Scorza, P., Stevenson, A., Canino, G., Mushashi, C., Kanyanganzi, F., Munyanah, M., & Betancourt, T. (2013). Validation of the "World Health Organization Disability Assessment Schedule for children, WHODAS-Child" in Rwanda. PloS one, 8(3), e57725. <https://doi.org/10.1371/journal.pone.0057725>
54. Reilly, M. C., Zbrozek, A. S., & Dukes, E. M. (1993). The validity and reproducibility of a work productivity and activity impairment instrument. PharmacoEconomics, 4(5), 353–365. https://doi.org/10.2165/00019053-199304050-00006
55. Chasens, E. R., Ratcliffe, S. J., & Weaver, T. E. (2009). Development of the FOSQ-10: a short version of the Functional Outcomes of Sleep Questionnaire. *Sleep, 32*(7), 915-919. doi:10.1093/sleep/32.7.915
56. Mundt, J. C., Marks, I. M., Shear, M. K., & Greist, J. H. (2002). The Work and Social Adjustment Scale: a simple measure of impairment in functioning. The British journal of psychiatry : the journal of mental science, 180, 461–464. https://doi.org/10.1192/bjp.180.5.461
57. Hahn, E. A., DeWalt, D. A., Bode, R. K., Garcia, S. F., DeVellis, R. F., Correia, H., et al. (2014). New English and Spanish social health measures will facilitate evaluating health determinants. Health Psychology, 33(5), 490–499.
